# Electronic Structure and *I*-*V* Characteristics of InSe Nanoribbons

**DOI:** 10.1186/s11671-018-2517-2

**Published:** 2018-04-18

**Authors:** A-Long Yao, Xue-Feng Wang, Yu-Shen Liu, Ya-Na Sun

**Affiliations:** 1 Jiangsu Key Laboratory of Thin Films, College of Physics, Optoelectronics and Energy, Soochow University, 1 Shizi Street, Suzhou, 215006 China; 2 Key Laboratory of Terahertz Solid-State Technology, Chinese Academy of Sciences, 865 Changning Road, Shanghai, 200050 China; 3 College of Physics and Engineering, Changshu Institute of Technology, Changshu, 215500 China

**Keywords:** InSe monolayer nanoribbon, Electronic structure, Negative differential resistance, Semiconductor-metal transition

## Abstract

We have studied the electronic structure and the current-voltage (*I-V*) characteristics of one-dimensional InSe nanoribbons using the density functional theory combined with the nonequilibrium Green’s function method. Nanoribbons having bare or H-passivated edges of types zigzag (Z), Klein (K), and armchair (A) are taken into account. Edge states are found to play an important role in determining their electronic properties. Edges Z and K are usually metallic in wide nanoribbons as well as their hydrogenated counterparts. Transition from semiconductor to metal is observed in hydrogenated nanoribbons HZZH as their width increases, due to the strong width dependence of energy difference between left and right edge states. Nevertheless, electronic structures of other nanoribbons vary with the width in a very limited scale. The *I-V* characteristics of bare nanoribbons ZZ and KK show strong negative differential resistance, due to spatial mismatch of wave functions in energy bands around the Fermi energy. Spin polarization in these nanoribbons is also predicted. In contrast, bare nanoribbons AA and their hydrogenated counterparts HAAH are semiconductors. The band gaps of nanoribbons AA (HAAH) are narrower (wider) than that of two-dimensional InSe monolayer and increase (decrease) with the nanoribbon width.

## Background

Atomically thin two-dimensional (2D) materials have attracted intensive interest in the last decade due to their unique electronic properties and promising application potential [1–4] mainly originated from their reduced dimensionality. One-dimensional (1D) nanoribbons can then be fabricated by tailoring the 2D materials [5] or assembling atoms precisely in the bottom-up way [6, 7]. In the nanoribbons, the electronic properties are further modulated by additional confinement and possible edge functionalization [8, 9]. For example, their energy gap, a key parameter of semiconductor, may be continuously adjusted by their width [10–15]. The dangling bonds of the edge atoms can be passivated by H atoms in proper environment, and the hydrogenation may stabilize the edges from structural reconstruction [16, 17].

Recently, a new member, the InSe monolayer, has been added to the 2D materials. Bulk InSe belongs to the family of layered metal chalcogenide semiconductors and has been intensively studied in the last decades [18–22]. Each of its quadruple layers has a hexagonal lattice that effectively consists of four covalently bonded Se–In–In–Se atomic planes. The quadruple layers are stacked together by van der Waals interactions at an interlayer distance around 0.8 nm. The stacking style defines its polytypes such as β, γ, and ε, among which the β and γ ones have direct band gaps. Nevertheless, the single quadruple InSe layer was successfully fabricated only in the last years by the mechanical exfoliation method [23, 24]. Since then, the observed extraordinary high electron mobility and special physical properties of InSe monolayers have triggered extensive study on their possible applications in optoelectronic devices [24–26] and electronic devices [27, 28]. For the sake of exploring novel functional properties, theoretical study can also be an efficient approach. Numerical simulations of structural, electric, and magnetic properties of InSe monolayers and their modulation by doping, defect, and, adsorption have been carried out [29–38]. The band structures of mono- and few-layer InSe have been carefully studied by density functional theory [29]. The dominant intrinsic defects in InSe monolayer have been figured out [30], and the properties of native defects and substitutional impurities in monolayer InSe have been estimated by calculation of formation and ionization energies [31]. In addition, it has been predicted that substitutional doping of As atoms can transfer InSe monolayer from nonmagnetic semiconductor to magnetic semiconductor/metal or half-semimetal [32]. The thermal conductivity of InSe monolayers can be greatly modulated by their size [33]. However, to our best knowledge, there are few studies on electronic properties of one-dimensional nanoribbons of InSe monolayer up to now.

In this paper, we carry out first-principles simulation on electronic properties of 1D bare zigzag, armchair, and Klein monolayer InSe nanoribbons and their hydrogen-passivated counterparts. Our studies indicate the transition from semiconductor to metal in hydrogen-passivated InSe zigzag nanoribbons and the interesting energy gap change in armchair nanoribbons. The current-voltage curves show diversified electric properties for nanoribbons with different edges.

## Methods

**Fig. 1:**
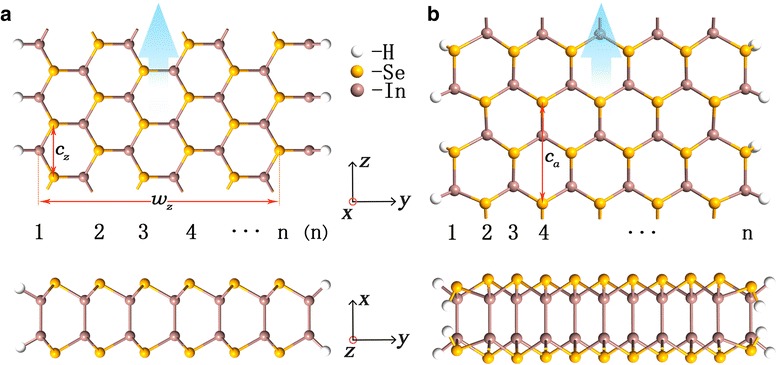
Top and side views of 6-HZKH (**a**) and 11-HAAH (**b**) InSe nanoribbons. Nanoribbon width number *n*, width *w*_*z*_, and lattice constants *c*_*z*_ or *c*_*a*_ are marked

The three typical edge patterns of honeycomb lattice, zigzag (Z), armchair (A), and Klein (K) are taken into account [39]. As illustrated in Fig. 1, a nanoribbon can be identified by its width number *n* and the combination of the types of its two edges. There are five classes of bare nanoribbons: *n*-ZZ, *n*-AA, *n*-KK, *n*-ZK, and *n*-KZ. Note that *n*-ZK is different from *n*-KZ because we assume that the left (right) Z edge ends with In (Se) atoms. If each edge atom is passivated by one hydrogen atom, we denote the passivated nanoribbons as *n*-HZZH, *n*-HAAH, *n*-HKKH, *n*-HZKH, and *n*-HKZH, respectively. A Se-In-In-Se quadruple layer of lattice constant 4.05 Å with Se-In layer distance 0.055 Å and In-In layer distance 0.186 Å is used to make nanoribbons before geometry optimization [21].

All the computations are performed using the Atomistix ToolKit (ATK) based on DFT with the pseudopotential technique. The exchange correlation functional in the local spin density approximation with the Perdew–Zunger parameterization (LSDA-PZ) is adopted. The wave functions are expanded on a basis set of double-ζ orbitals plus one polarization orbital (DZP). An energy cutoff of 3000 eV, a *k*-space mesh grid of 1 × 1 × 100, and an electronic temperature of 300 K are used in the real-axis integration for the non-equilibrium Green’s functions. A 15-Å thick vacuum layer in the supercells is adopted to separate the nanoribbons from their neighbor images in both *x* and *y* directions and to ensure the suppression of the coupling between them. Band structures are calculated after full geometry relaxation with a force tolerance of 0.02 eV/Å^−1^.

To simulate the electronic transport property of the nanoribbons, we connect each one into a circuit with left (right) chemical potential *μ*_*L*_(*μ*_*R*_) [40, 41]. The nanoribbon can then be partitioned into three regions, the left (right) electrode L (R) and the central region C. The spin-dependent current can be estimated by the Landauer-Büttiker formula [42].

$$ {I}_{\sigma}\left({V}_b\right)=\frac{e}{h}{\int}_{-\infty}^{+\infty }{T}_{\sigma}\left(E,{V}_b\right)\left[{f}_L\left(E-{\mu}_L\right)-{f}_R\left(E-{\mu}_R\right)\right] dE $$

with spin *σ* =  ↑ , ↓ and voltage bias *V*_*b*_ = (*μ*_*R*_ − *μ*_*L*_)/*e*. Here, ${T}_{\sigma}\left(E,{V}_b\right)= Tr\left[{\Gamma}_L{G}_{\sigma }{\Gamma}_R{G}_{\sigma}^{\dagger}\right]$ is the transmission spectrum with *G*_*σ*_ the retarded Green’s function in region C and Γ_*L*_ (Γ_*R*_) the coupling matrix between C and L (R). *f*_*L*_ (*f*_*R*_) is the Fermi distribution function of electrons in L (R).

## Results and Discussion

In Fig. 1, we scheme the top and side views of (a) 6-HZKH and (b) 11-HAAH nanoribbons with lattice constants *c*_*z*_ = 4.05 Å and *c*_*a*_ = 7.01 Å, respectively. Edge K is along the direction parallel to that of edge Z. The extending direction *z* of the nanoribbon is marked by blue arrows. Different from the case in graphene nanoribbon [39], no edge reconstruction is observed for the three edge styles in both bare and H-passivated InSe nanoribbons, and our simulation indicates that they are all energetically stable.

**Fig. 2:**
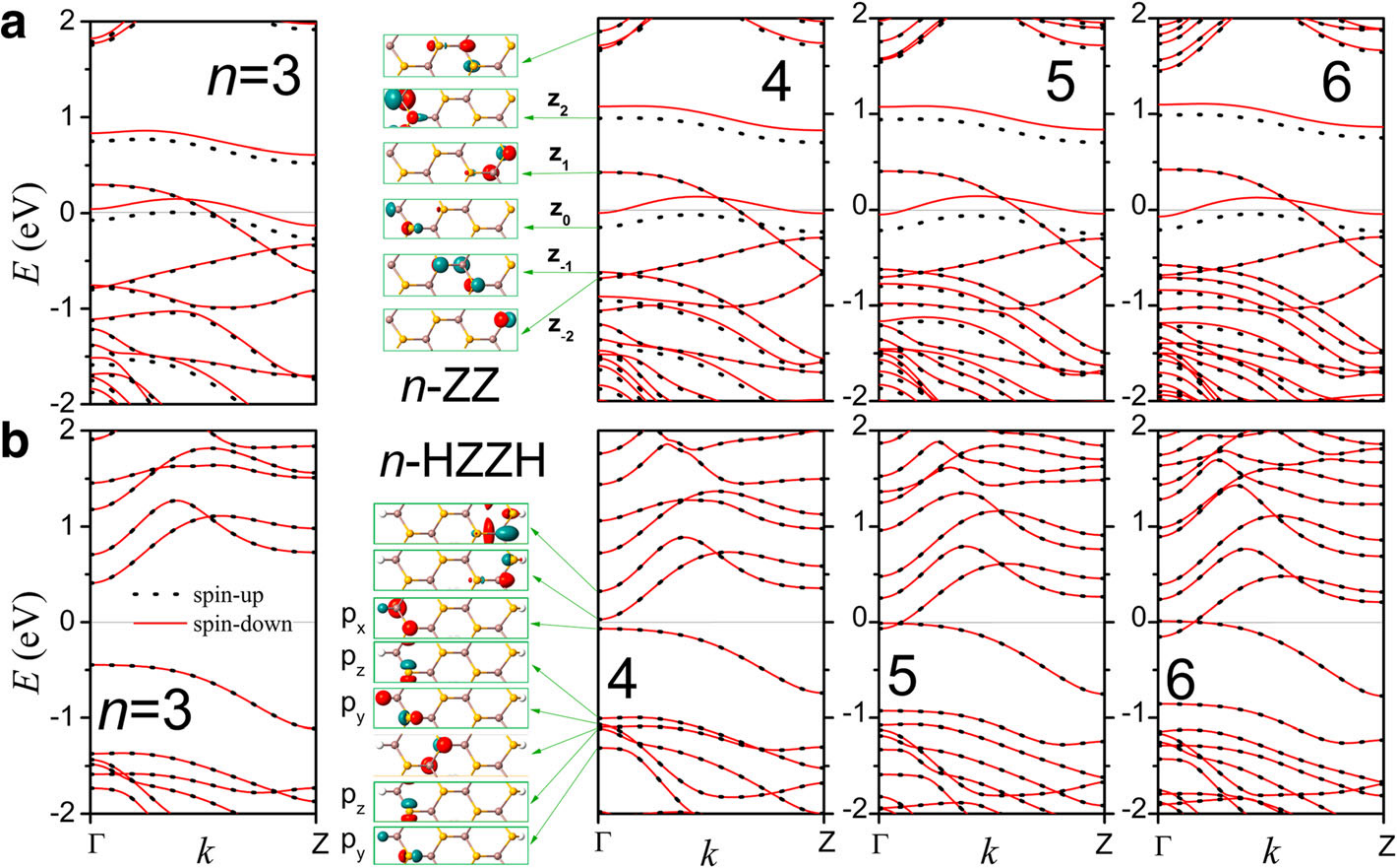
The band structures of **a** 3-, 4-, 5-, and 6-ZZ nanoribbons and **b** 3-, 4-, 5-, and 6-HZZH nanoribbons. Г-point Bloch states near the Fermi energy are shown for *n* = 4. The orbits of the states below the Fermi energy are indicated for 4-HZZH nanoribbon

Bare *n*-ZZ nanoribbons are magnetic metal except the 2-ZZ one which has a reconstructed geometry and appears semiconductor. They have similar band structures as illustrated in Fig. 2a. The *p* orbitals of edge Se atoms dominate the contribution to the states near the Fermi energy similar to the case of InSe monolayer [32], but more contributions from the In atoms are observed here. The two partially occupied bands are from the left and right edge states, respectively, as shown by the Г-point Bloch states for 4-ZZ nanoribbon. One of them is spin split and a net magnetic moment, e.g., 0.706 μ_B_ for 4-ZZ nanoribbon, appears in each primitive cell on the left edge.

When the edge atoms are passivated by H atoms, *n*-HZZH nanoribbons become nonmagnetic semiconductor for *n* = 3, 4 and metal for *n* > 4 as shown in Fig. 2b. Note that the structure becomes unstable for *n* = 2. In 4-HZZH nanoribbon, the Bloch states at Г in conduction (valence) bands near the Fermi energy are confined to the right (left) edge. They have components similar to those in 2D InSe monolayer except the H atomic orbital parts. The highest five bands of the left edge states are composed of one *p*_x_, two *p*_y_, and two *p*_z_ orbitals of Se edge atoms. The energy bands of the right (left) edge states are similar to the conduction (valence) bands in the Γ-K direction of 2D InSe monolayer [32]. Their separation in energy depends strongly on *n* though their dispersions are insensitive to *n*. We define *E*_d_ as the energy difference between the minimum of the right edge states and maximum of the left edge states.

**Fig. 3:**
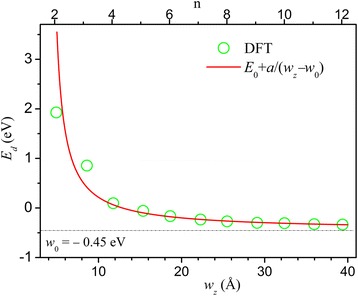
The minimal energy differences *E*_d_ between the right and left edge states near the Fermi energy in *n*-HZZH nanoribbons are shown versus *n* and *w*_*z*_. The fit curve is in red

In Fig. 3, we plot *E*_d_ versus *n* and *w*_z_ and found approximately an inverse dependence *E*_*d*_ ≈ *E*_0_ + *a*/(*w*_*z*_ − *w*_0_) with *E*_0_ =  − 0.45eV, *w*_0_ = 4Å, and *a* = 4eVÅ. This behavior is similar to the width dependence of energy gap in zigzag graphene and B-N nanoribbons [12–15, 43–47] having origin of electron-electron interaction. Narrow HZZH InSe nanoribbons are semiconductors, and a transition from semiconductor to metal occurs as the width increases.

**Fig. 4:**
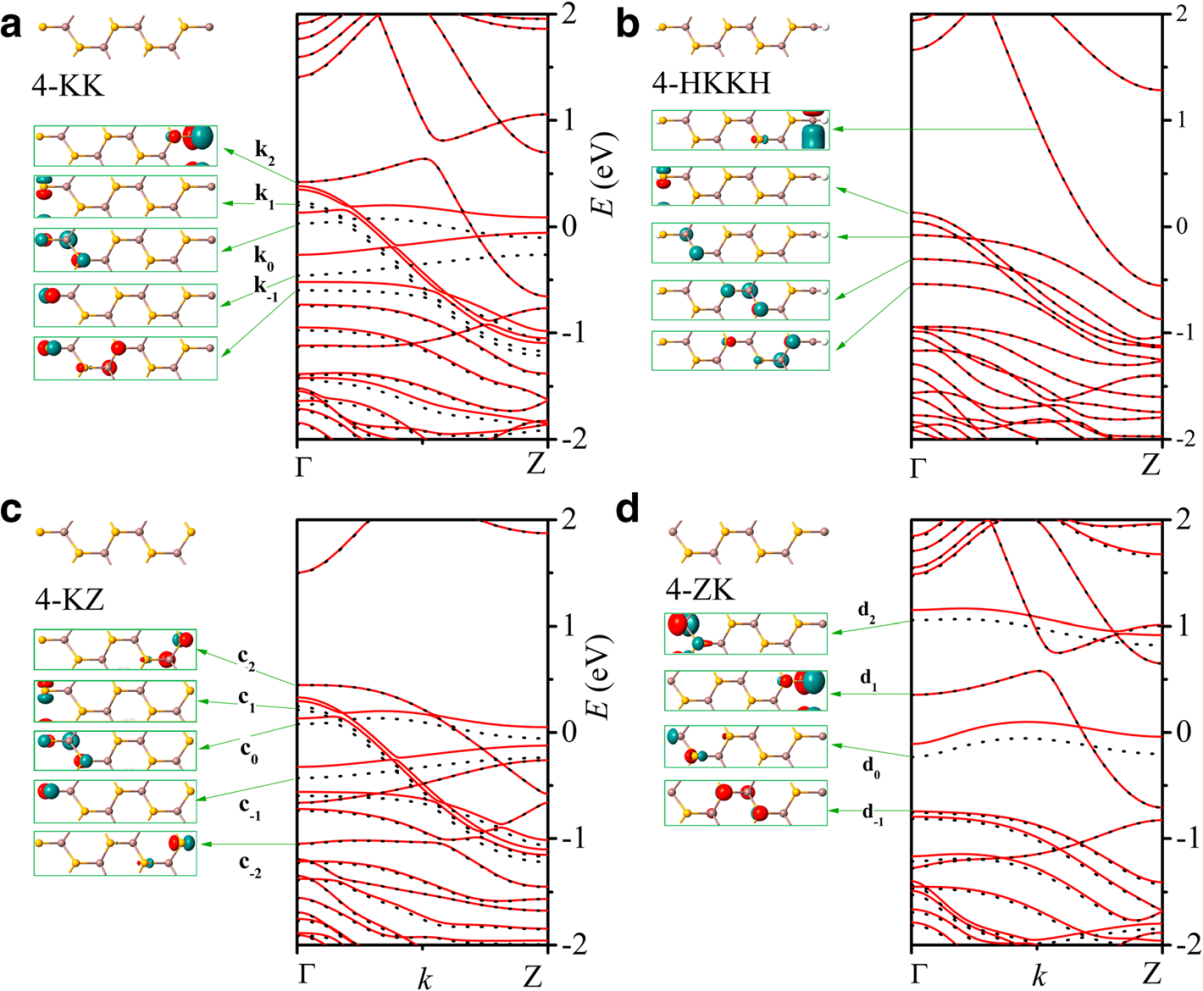
The band structures and Γ-point Bloch states of 4-KK (**a**), 4-HKKH (**b**), 4-KZ (**c**), and 4-ZK (**d**) nanoribbons

The band structures of *n*-KK and *n*-HKKH nanoribbons are not sensitive to the width number *n* as exemplified in Fig. 4a, b, respectively, for *n* = 4. Compared to zigzag edge, bare Klein edge has more dangling bonds which results in significant change of the band structure. Orbitals of edge Se atoms usually have lower energy than those of edge In atoms, similar to ZZ nanoribbon. In HKKH nanoribbons, the suppression of the *p* orbital of edge In atoms and the *p* orbital of edge Se atom by the passivation of H atoms is obvious. Nevertheless, one H atom is not enough to passivate all the dangling bonds of the edge atoms. Both KK and HKKH nanoribbons are metal.

In nanoribbons with a mixing of zigzag and Klein edges, we observe a combination of energy bands of the two kinds of edges near the Fermi energy. As shown in Fig. 4c for the 4-KZ nanoribbon, the dispersion and Γ-point Bloch states of bands c_1_, c_0_, and c_−1_ are the same as those of band k_1_, k_0_, and k_−1_ in 4-KK nanoribbon as plotted in Fig. 4a, while bands c_2_ and c_−2_ are the same as band z_1_ and z_−2_ of 4-ZZ nanoribbons in Fig. 2a. Similarly, the band structure of the 4-ZK nanoribbon, as illustrated in Fig. 4d, is composed of band d_1_ from the right Klein edge and bands d_2_, d_0_, and d_−1_ from the left zigzag edge. Since *n*-ZK and n-KZ nanoribbons keep part of the energy bands of *n*-KK nanoribbons near the Fermi energy, they are both metal as the *n*-KK nanoribbons. For the same reason, the H-passivated nanoribbons mixing edges Z and K are also metallic.

**Fig. 5:**
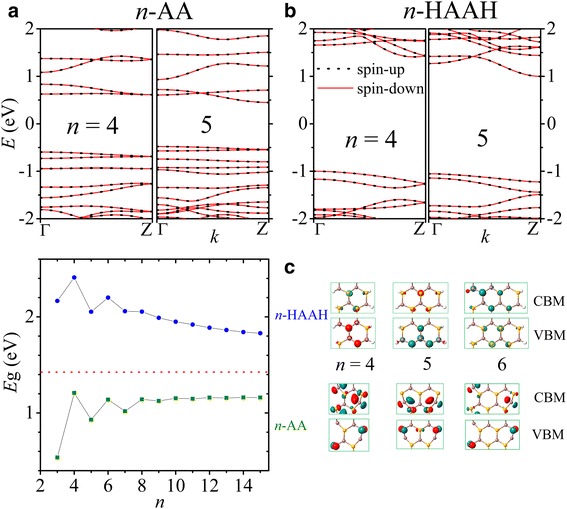
The band structures of 4- and 5-AA nanoribbons are shown in **a** and those of 4- and 5-HAAH in **b**. The energy gaps *E*g of *n*-AA (green) and *n*-HAAH (blue) nanoribbons are plotted versus *n* in **c** with the gap of InSe monolayer (red) marked. The Bloch states at CBM and VBM for *n* = 4, 5, and 6 are shown in the right panels of **c**

Both the AA and HAAH nanoribbons are nonmagnetic semiconductors as shown in Fig. 5a, b, where the band structures are plotted for *n* = 4, 5. The passivation of H atoms can improve the structural stability energetically and enlarges the energy gap. Interestingly, the energy gap has a zigzag dependence on the nanoribbon width, showing an odd-even family-like behavior as in graphene and B-N nanoribbons [10–15, 43–47]. As illustrated in Fig. 5c, *n*-AA nanoribbons have a gap (olive square) narrower than that of 2D InSe monolayer (red dash). The gap increases (decreases) monotonically with the width for odd (even) *n* and converges to a value of 1.15 eV at the large width limit when the two edges are decoupled from each other and stable their energy [13]. The Bloch states of valence band maximum (VBM) at Г point and conduction band minimum (CBM) at Z point are also shown in Fig. 5c. The parity behavior is observed again with the symmetric (*n* = 5) or diagonal (*n* = 4, 6) distribution of the states around edge Se atoms at VBM and around edge In atoms at CBM.

On the other hand, the gaps of *n*-HAAH nanoribbons (blue circle) are wider than their 2D counterpart and decrease with the width for both odd and even *n*. In passivated nanoribbons, the Bloch states at VBM and CBM have much less edge component. The corresponding energy gaps are about 1 eV wider than those of the bare nanoribbons, and the difference diminishes with width increase [13].

**Fig. 6:**
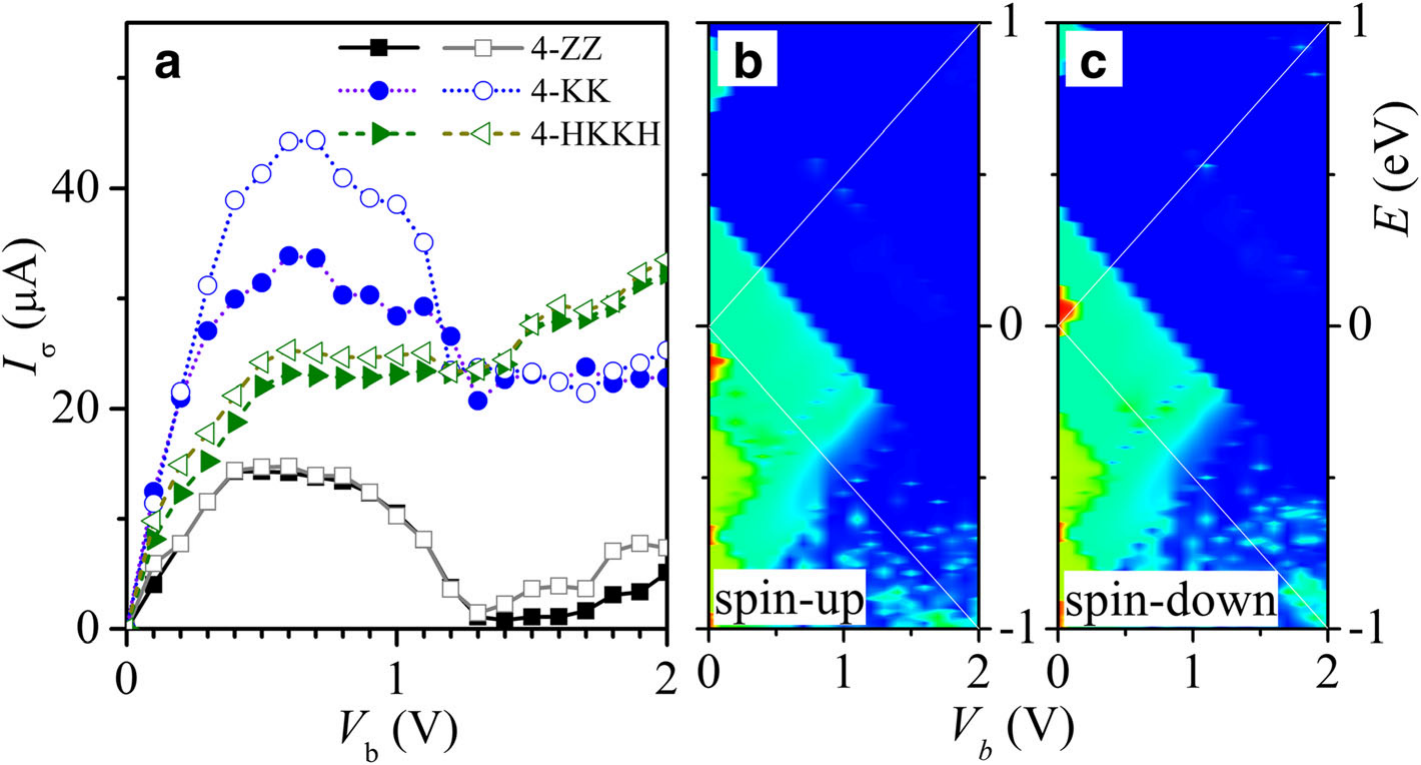
**a** The spin-up (filled) and spin-down (empty) *I*-*V* characteristics of 4-ZZ (square), 4-KK (circle), and 4-HKKH (triangle) InSe nanoribbons are presented. The corresponding transmission spectra of spin-up (**b**) and spin-down (**c**) are shown for the 4-ZZ nanoribbon. The transport window between *μ*_*L*_ and *μ*_*R*_ is marked by the white lines

In Fig. 6a, we show the current-voltage (*I*-*V*) characteristic of above metallic InSe nanoribbons 4-ZZ (square), 4-KK (circle), and 4-HKKH (triangle). Spin-up (spin-down) curves are marked by filled (empty) symbols. The Landauer-Büttiker formula has been employed to calculate the spin dependent current *I*_*σ*_ when a voltage bias *V*_b_ is applied between electrodes L and R, with *μ*_*R*_ = *eV*_*b*_/2 and *μ*_*L*_ =  − *eV*_*b*_/2 assumed. Negative differential resistance (NDR) and spin polarization are observed in 4-ZZ and 4-KK bare nanoribbons under a bias in the region between 0.5 and 1.2 V. The peak-to-valley ratio of NDR is larger than 10 for the 4-ZZ nanoribbon due to the transversal mismatch of wave functions among energy bands near the Fermi energy as illustrated in Fig. 2a and explained in the following. Band z_1_ is the dominant transport channel under *V*_b_ < 1.2 V as indicated by the spin-up and spin-down transmission spectra in Fig. 6b, c, respectively. However, the wave functions of band z_1_ are orthogonal to or are separated in space from those of nearby bands z_2_, z_−1_, and z_−2_. This leads to the mismatch between the states z_1_ in one electrode and those of the same energy in the other electrode under *V*_b_. The electrons from band z_1_ in one electrode then have difficulty to transport to the other electrode with energy conservation. As a result, the *I-V* curve of nanoribbon 4-ZZ shows a single-band characteristic with strong NDR. Furthermore, the spin split of band z_0_ leads to the spin polarization in the linear regime. In the passivated 4-HKKH nanoribbon, however, the current saturates in the above NDR bias region.

## Conclusions

We have systematically investigated the electronic properties of InSe nanoribbons with Z, A, or K edges. The edges play a key role in determining the properties since electron states near the Fermi energy have big weight of edge atomic orbitals. Bare Z and K edges are conductive and magnetic. Strong edge-edge interaction may lead to the transition of *n*-HZZH nanoribbons from semiconductor to metal as *n* increases. As a result, bare and H-passivated nanoribbons with Z and K edges are metallic except very narrow ones. *n*-AA and *n*-HAAH are nonmagnetic semiconductors with energy gaps narrower and wider, respectively, than that of InSe monolayer. Their gaps approach each other in a zigzagged way as *n* increases, showing an even-odd behavior. The current-voltage curves of ZZ and KK nanoribbons are characterized by strong single-band NDR and spin polarization.

## Abbreviations

1D: One-dimensional
2D: Two-dimensional
A: Armchair
CBM: Conduction band minimum
K: Klein
VBM: Valence band maximum
Z: Zigzag
